# Emergency vaccination of cattle against lumpy skin disease: Evaluation of safety, efficacy, and potency of MEVAC^®^ LSD vaccine containing Neethling strain

**DOI:** 10.1007/s11259-022-10037-2

**Published:** 2022-12-03

**Authors:** Abdel-Hamid Bazid, Momtaz Wasfy, Mohamed Fawzy, Mohamed Nayel, Mohamed Abdelmegeid, Randa Y. Thabet, Hui Sian Yong, Magdy M. El-Sayed, Asmaa Magouz, Yassien Badr

**Affiliations:** 1 Department of Virology, Faculty of Veterinary Medicine, University of Sadat City, 32958 Menoufiya, Egypt; 2 Middle East for Vaccines (MEVAC®), 44813 Sharquia, Egypt; 3 Department of Virology, Faculty of Veterinary Medicine, Suez Canal University, 41522 Ismailia, Egypt; 4 Department of Medicine and Infectious Diseases, Faculty of Veterinary Medicine, University of Sadat City, Menoufiya, Egypt; 5 Department of Animal Medicine, Faculty of Veterinary Medicine, Kafrelsheikh, University, Kafr El Sheikh, Egypt; 6 Senior Regional Business Manager, Asia Kemin Biologics®, 12 Senoko Drive, 758200 Singapore, Singapore; 7 Department of Virology, Faculty of Veterinary Medicine, Kafrelsheikh University, 33516 Kafrelsheikh, Egypt; 8 Department of Animal Medicine (Branch of Infectious Diseases), Faculty of Veterinary Medicine, Damanhour University, 22511 El‑Beheira, Egypt

**Keywords:** Lumpy skin disease, Vaccine, Neethling strain, Seroconversion, Post-vaccination reaction, Vaccine evaluation

## Abstract

Lumpy skin disease (LSD) is an emerging disease of cattle causing significantly high economic losses. Control of LSD depends on the use of homologous attenuated LSD virus strains isolated originally from South Africa (the Neethling strain). The virus belongs to the genus *Capripoxvirus*, which includes sheep pox virus and goat pox virus. The present study was conducted to evaluate the safety and efficacy of a new live attenuated LSD vaccine produced by Middle East for Vaccines (MEVAC^®^) based on the Neethling strain. Tests were performed both in Egypt and Vietnam. Safety was evaluated by inoculation of five cattle with 10 times the recommended dose and observation of the animals for 14 days. Immunogenicity was tested at different periods post-vaccination (PV) in animals receiving the recommended doses of the vaccine using ELISA and virus neutralization test. Five cows were used to determine the protection index (PI) and non-vaccinated control cattle were included. Three calves were challenged by intradermal inoculation of the wild virus (5 × 10^5^ TCID_50_) 28 days PV. Field or mass vaccination experiments were conducted in Vietnam during national campaigns in the summer of 2021 with 4301 vaccinated animals closely monitored after vaccination. In the field, around 2% (80/4301) of the animals showed hyper-reactivity, and 0.6% (24/4301) showed small skin swellings that disappeared within few hours PV. Abortion was recorded in three animals (0.3% 3/867). Challenged animals were resistant to clinical disease and PI value was 3.5 log_10_. Meanwhile, antibody levels determined by the ELISA were inconsistent among animals and laboratories during the study period. Overall, the findings point to a new safe and effective LSD vaccine.

**Supplementary Information:**

The online version contains supplementary material available at 10.1007/s11259-022-10037-2.

## Introduction

Lumpy skin disease (LSD) is a vector-borne transboundary pox disease of cattle characterized by fever, lethargy, eruption of numerous skin nodules, lameness, impaired appetite, decreased milk output, and systemic lymphadenitis (Tuppurainen et al., 2017; Tuppurainen and Oura, 2012). The nodules are usually round, slightly raised, firm, painful and may rupture and become infected. The disease is caused by the lumpy skin disease virus (LSDV), a member of family *Poxviridae* and the genus *Capripoxvirus* which is a double-stranded DNA virus (Lefkowitz et al., 2018). Infection is caused by a virus endemic in large parts of the world, including most of the African countries, the Middle East, and Asia, posing a transmission risk to countries which have not yet had LSDV outbreaks (Cornell et al., 2017; Namazi and Khodakaram Tafti, 2021). Outbreaks in Europe have been controlled and eradicated through vaccination (Calistri et al., 2020).

LSD is recognized as one of the greatest threats to dairy and meat farms in Africa, the Middle East and Asia. Because of its transboundary nature and the considerable socioeconomic consequences of skin degradation, reduced milk output, abortions, low weight gain, temporary or permanent infertility, and secondary bacterial infections, the disease has been designated as a notifiable disease by the World Organization for Animal Health (WOAH) (previously named: Office international des epizooties (OIE)) (WOAH, 2022b). Furthermore, in the case of an outbreak, costly control and eradication procedures are needed (Wolff et al., 2020). Besides having a major impact on national output, an outbreak causes a limitation in live animals and animal products global trading (Farra et al., 2022).

Globally, the African countries were the first to encounter LSD outbreaks, but the disease has since extended to the Middle East (though not reported in Morocco, Algeria, Tunisia, Libya), Asia, and more lately, Europe (Molini et al., 2018; Rouby et al., 2021; Tuppurainen and Oura, 2012). The disease was suspected to be spread out of Africa into Israel in August 1989, via a wind-borne route of transmission by the *Stomoxys calcitrans* fly. This notion was explained by the fact that no new animals were admitted into affected herds, and that LSDV has previously been isolated from stable flies collected while feeding on the infected cattle (Paslaru et al., 2021). In Asia, the first report of LSD was in 2019 in China and the virus moved to Vietnam and nearby countries in 2020 (Roche et al. 2020; Tran et al., 2021). Young calves, lactating cows and underweight animals are the most vulnerable due to compromised immune systems (Namazi and Khodakaram Tafti, 2021). In Egypt, the disease was first identified in 1988 affecting cattle in the northeastern region (Ismailia province) upon livestock importation from Somalia (Ali et al., 1990; House et al., 1990). After 17 years, LSD reemerged in Egypt in 2006 in a major outbreak after the entry of infected cattle imported from Ethiopia, causing serious economic losses to livestock industry (Awadin et al., 2011; Salib and Osman, 2011; Sharawi and Abd El-Rahim, 2011). Those economic losses ranged between 147 and 539 EUR per animal in affected herds in some European countries during 2016 (Casal et al., 2018). Despite widespread vaccination, outbreaks were reported in Egypt in, 2011, 2014, 2017, 2018, and 2020 (Badr et al., 2022; Allam et al., 2020; El-Tholoth and El-Kenawy, 2016; Elhaig et al., 2017; Rouby et al., 2019; Rouby et al., 2021; Shalaby et al., 2016) .

Since sheep pox virus (SPV) and goat pox virus (GPV) belonged to the genus *Capripoxvirus*, they were cross immunogenic for LSD, but protection of sheep and goat by LSDV against these viruses has not been reported to our knowledge. However, Kenya LSDV strain (long thought to be a sheep and goat pox strain) has been extensively used to protect small ruminants against their respective diseases with inconsistent results (Hamdi et al., 2020). Now that the attenuated sheep or goat pox viruses have been used as heterologous vaccines, with some success, some Neethling-based LSD vaccines showed severe post-vaccination (PV) reactions and vaccination failure. Partial protection of cattle against LSD has been achieved in Egypt by an attenuated Romanian strain of SPV vaccine (Gaber et al., 2022). While it has been reported that vaccination of cattle with LSDV resulted in the appearance of antibodies in 50% of the animals after three weeks, no antibody response was detected with SPV vaccines (Hamdi et al., 2020). In addition, heterologous vaccines sometimes failed due to the appearance of LSD symptoms in some vaccinated animals, raising doubt about their field efficacy (Abutarbush et al., 2015; Brenner et al., 2009). This has been attributed to the nature of some cattle breeds or type of the vaccine used, since heterologous ones are administered at 10-fold higher concentrations than homologous LSD strains (Abdallah et al., 2018; Bamouh et al., 2021). Of the homologous LSD strains, a Neethling strain isolated in South Africa was successfully attenuated and used (Davies, 1991). The vaccine proved innocuous and immunogenic, though some local reactions have been observed in some animals. The vaccine was used in six Balkan countries during 2016–2017 (Bulgaria, Greece, Serbia, Montenegro, Former Yugoslav Republic of Macedonia, and Albania) and the average ratio of its effectiveness was 79.8% (range = 62.5–97%) (Klement et al., 2020). The discrepancies and inconsistency of results obtained from many heterologous and some homologous vaccines against LSD prompted some veterinary vaccines-based corporations to produce a homologous vaccine based on the Neethling strain (MEVAC^®^ live attenuated LSD vaccine). The safety and effectiveness of this product were evaluated in Egypt and Vietnam during an emergency season between 2020 and 2021.

## Materials and methods

### LSD vaccine

The LSD vaccine used in the current study is a live attenuated LSDV vaccine based on an isolate that was isolated, characterized and attenuated in Egypt. Partial nucleotide sequencing showed that the isolate is genetically related to the reference LSDV Neethling strain (ScienSano report; Supplementary files 1 and 2). The vaccine is manufactured by the Middle East for Vaccines (MEVAC^®^) by propagation of the virus on Madin-Darby bovine kidney (MDBK) cells or fetal lamb heart cells. Extensive reversion to virulence studies have been conducted during vaccine development and vaccine samples were sent to ScienSano for evaluation according to their method (Agianniotaki et al. 2017). The vaccine is prepared in one dosage form containing10^3.5^ log_10_ TCID_50_/dose.

The sterile diluent used for vaccine reconstitution is a sterile phosphate-buffered saline solution. The reconstituted vaccine is kept on ice and should be protected from light until use. The reconstituted vaccine should be used within two hours.

### Experimental studies

The experimental studies were conducted using Holstein Friesian cows to determine the safety, efficacy, and potency of the examined vaccine.

#### Safety

Safety of MEVAC^®^ LSD vaccine was experimentally evaluated in dairy cows that did not have antibodies against LSDV by ELISA or serum neutralization tests (SNT). The cattle were maintained in a large animal unit with a high level of containment (BSL-3) at the Central Laboratory for Evaluation of Veterinary Biologics (CLEVB, Cairo, Egypt). Five vials of the lyophilized vaccine were randomly chosen, reconstituted in sterile diluent, and pooled. Five pregnant cows were inoculated subcutaneously (S/C) with 10 times the recommended dose of the vaccine (10 ml) divided into two injection sites. Two animals were inoculated with the vaccine diluent and used as the control. All animals were clinically inspected daily for any adverse vaccine reactions for two weeks PV. The experiment was conducted in both Egypt and Vietnam before vaccine use.

In Vietnam, a pilot experiment was conducted to examine safety of the vaccine and its effects on the general health status of the animals before the expanded large scale (field) vaccination. In this experiment, 65 animals (25 pregnant heifers, 20 lactating cows, and 20 pregnant dry cows) were inoculated by the in-label dose of the vaccine (1 ml containing 10^3.5^ log_10_ TCID_50_). The animals were clinically inspected for any adverse vaccine reactions. Further, main physiological parameters including rumination index, health index, and milk yield were monitored as described previously (Stangaferro et al., 2016; Vanhoudt et al., 2015). Rumination index and health index were automatically monitored by specialized software for cow health management (SCR-Israel). The health index scale ranged between 0 and 100 units and a reading < 86 on at least one day within 2–5 days PV was regarded as a health disorder. Milk yield was monitored by DeLaval DelPro™ farm system (Sweden). Serum samples were also collected from vaccinated animals on days 21, 28, 35 and 42 PV for the antibody testing by using the ID Screen Capripox Double Antigen ELISA kit (ID.Vet, Montepellier, France), to examine vaccine potency and seroconversion percent.

#### Efficacy

Efficacy of the vaccine was examined by using two methods; first one is the protection index (PI) and the second one is the challenge test. The PI, which is the difference between the virulent virus titer (over log10^6^ TCID_50_) in vaccinated and non-vaccinated controls (WOAH, 2022b). The experiment was conducted at 28 days PV at the Central Laboratory for Evaluation of Veterinary Biologics (CLEVB, Cairo, Egypt). Six dilutions of the LSDV wild strain locally identified in Egypt (titer < 5 × 10^6^ TCID_50_) were inoculated intradermally (0.1 ml per inoculum) along the length of the flank in four replicates down the flank of five vaccinated and two non-vaccinated (control) cows. The animals were observed for the development of clinical signs at inoculation sites for 14 days post-inoculation and rectal temperature, general condition, and specific signs of LSD were recorded. However, hypersensitivity reactions that appeared within 24 hours (h) at the sites of inoculation were ignored as they quickly diminished. The virus titer was determined from LSD inoculated flanks of vaccinated and unvaccinated animals two weeks after inoculation. A difference between *in vivo* titrations of the virus in vaccinated and non-vaccinated animals ≥ log_10_ 2.5 is considered suitable for vaccine release (evidence of protection) (WOAH, 2022b).

The challenge experiment was conducted in Vietnam, as previously described (Wolff et al., 2020). Six calves (6–12 months old) were used. Three of them were vaccinated with the in-label dose and the other three animals were not vaccinated and used as a control. All animals were challenged after 28 days of vaccination with field LSDV (LSD/KN1/2020) isolated in Vietnam by intradermal injection of 5 × 10^6^ TCID_50_ of the virus (1 ml) at the neck. Animals were observed for clinical signs for two weeks after the challenge test.

#### Potency

Three small farms in Egypt (23 animals each) received one vaccine dose (1 ml containing 10^3.5^ log_10_ TCID_50_). The sera were collected at days 28, 45, 60, 120, and 150 PV to evaluate the antibody response by the IDvet ELISA following the manufacturer’s instructions. The readings were calculated as a percent of the sample optical density (OD) to the positive control OD (S/P %) as follows:

$$S/P \equiv \frac{{test\,sample\,OD - negative\,control\,OD}}{{positive\,control\,OD - negative\,control\,OD}} \times 100$$

Values < 30 were considered negative, and ≥ 30 were positive, according to the manufacturer’s recommendations.

Virus neutralization test (VNT) was also examined for the same sera. The test was performed in 96-microwell plates (Nunc, Thermo Fisher Scientific, USA) and titers were expressed as the logarithm (base 10) of the reciprocal of the last dilution of serum that neutralized 100× TCID_50_ of the virus in 50% of the wells (Krešić et al., 2020; WOAH, 2022a).

### Field study

In a large scale, the vaccine was used to immunize more than 3.5 million cattle during a national campaign in Egypt in the summer of 2021. In Vietnam, more than two million cattle were also vaccinated, with 4301 animals at two farms were closely monitored for protection percent (number of noninfected-vaccinated animals/total vaccinated animals X 100) after one year of vaccination. PV reactions including hyper-reactivity, skin swellings, and abortion were also monitored. In addition, 29 vaccinated animals from each farm were used in an additional experiment, in which serum samples were collected from each animal at days 21, 28, 35 and 42 PV to examine serocoversion by using Idvet ELISA. The sample of each animal was divided into three portions and sent to three different laboratories to assess the consistency of results.

### Statistical analysis

In this study, the statistical functions of Microsoft 365 were used in the estimation of central tendency measures, standard deviation, performance of the t-test, correlation coefficient, normal distribution p-values, and design of the tables and graphs (Microsoft Corporation, 2021).

**Table 1:** Mean rumination indices in animals from two farms (n = 65 animals) ± standard deviation (SD)

	Before vaccination	At vaccination	1 day PV^※^	2 days PV	3 days PV	4 days PV	5 days PV	6 days PV	7 days PV
Average	467.9	457.3	452.65	464.25	461.5	456.3	466.45	467.35	466.55
± SD	18.53	4.10	17.32	9.69	17.11	34.93	37.26	30.62	41.51

^※^PV: Post-vaccination.

## Results

### Experimental studies

#### Safety

In the safety experiment, despite the 10 times doses, MEVAC^®^ LSD vaccine was safe and all vaccinated cows showed normal body temperature (< 39 ^o^C), measured at 24 h PV. Pregnancy was not affected and no adverse reactions, abnormal clinical signs, or local reactions were observed. All five inoculated animals grew stably. When this experiment was repeated in Vietnam, one of the five animals showed shrunken skin at the vaccination site and tears, then spontaneously recovered within few hours. On the other hand, all uninoculated controls did not develop any symptoms during contact and remained seronegative during the observation period.

Genetically, the genome of the vaccine virus was differentiable from the wild-type LSDV and did not contain detectable parts of wild-type LSDV genome (ScienSano report; Supplementary file 1). Further, the vaccine virus was highly akin (≥ 95%) to those of the Neethling vaccine OBP KX76465, cro 216 MG 972,412, SIS lumpyvax KX 764,643, Neethling LW 1959 AF 409,138, Herbivac vaccine KX764644, LSDV 148-GP-RSA-1997 MN6 36,843, isolate 103-GP-1991 MN636839 and isolate LSD 220-2NW-RSA-1993 MN636842 (ScienSano report; Supplementary files 1 and 2).

**Table 2:** Mean health indices in two farms (n = 65 animals) ± standard deviation (SD)

	Before vaccination	At vaccination	1 day PV^※^	2 days PV	3 days PV	4 days PV	5 days PV	6 days PV	7 days PV
Average	108.1	94.9	108.0	95.0	95.05	94.55	120.9	95.35	95.7
± SD	17.82	1.13	15.84	2.40	0.49	0.78	36.49	1.34	1.13

^※^PV: Post-vaccination

**Fig. 1:**
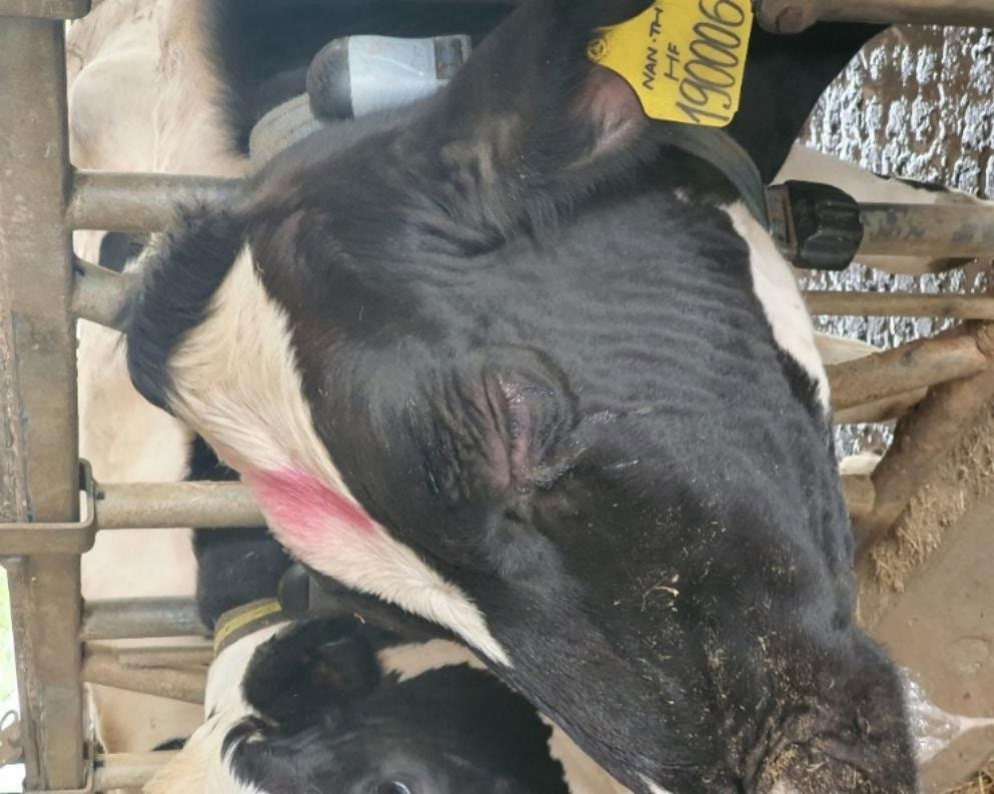
A cow’s head showing tears, ruffled hair, and wrinkled face skin after lumpy skin disease vaccination

Of the 65 animals closely observed in Vietnam after vaccination using the recommended dose, right after injection, one cow was found to have hyper-sensitivity reaction signs with some symptoms such as ruffled hair, facial wrinkles (Fig. 1), skin shrinkage at the injection site, and rapid respiration rate. The reacted cow gradually recovered without any complications. Two vaccinated cows showed small swellings in the skin, which gradually disappeared in about five h PV. Abortion didn’t occur in any of the pregnant animals used in this experiment.

**Fig. 2:**
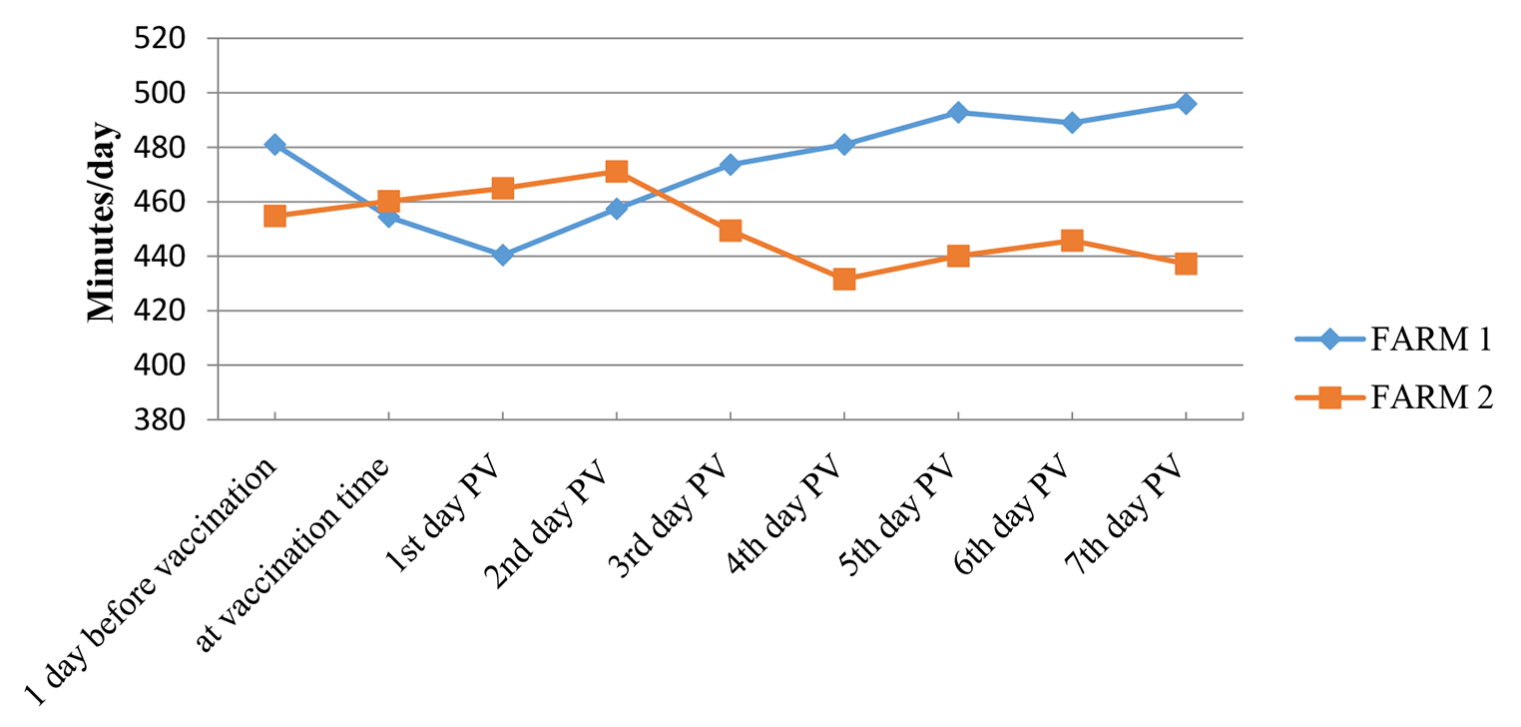
**Average rumination index monitored after vaccination in two cattle farms in Vietnam. Farm-1** (n = 30): average rumination index decreased by 1st day post-vaccination (PV), then recovered and tended to increase from 2nd day and remained stable afterwards. **Farm-2** (n = 35): average rumination index increased by 2nd day PV then decreased from 3rd to 4th days and was recovered afterwards

In one farm, rumination index slightly decreased after vaccination then returned to normal (444-449 minutes (min)) by the 3rd day PV. In the other farm, rumination index increased after vaccination to 464 min then gradually decreased after the 3rd day PV to reach 431 min. Statistically, the mean index values for the two farms were not significantly different during the first seven days PV (p = 0.6) as shown in Table 1; Fig. 2.

**Fig. 3:**
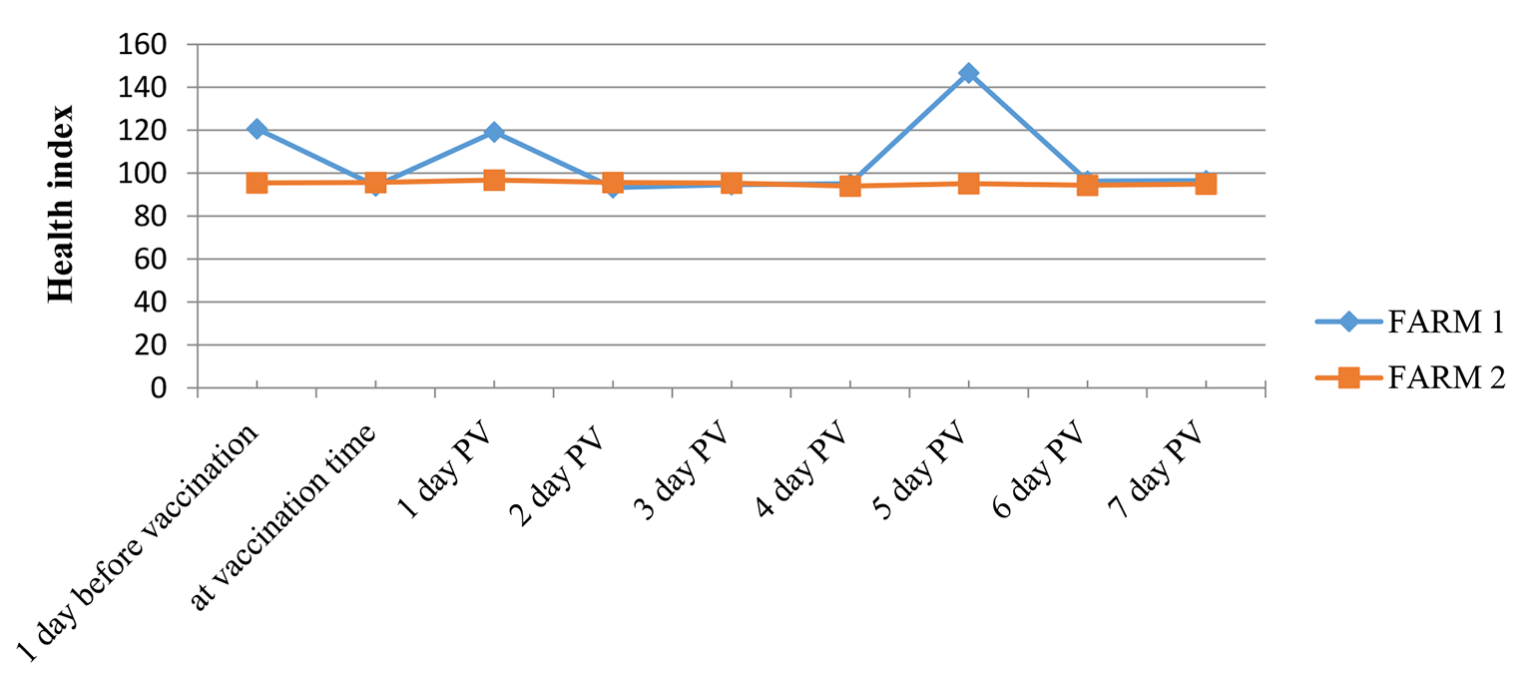
**Average health index monitored after vaccination in two cattle farms in Vietnam. Farm-1** (n = 30): showed minor fluctuations after vaccination. **Farm-2** (n = 35): average health index was stable after vaccination. PV: Post-vaccination

**Fig. 4:**
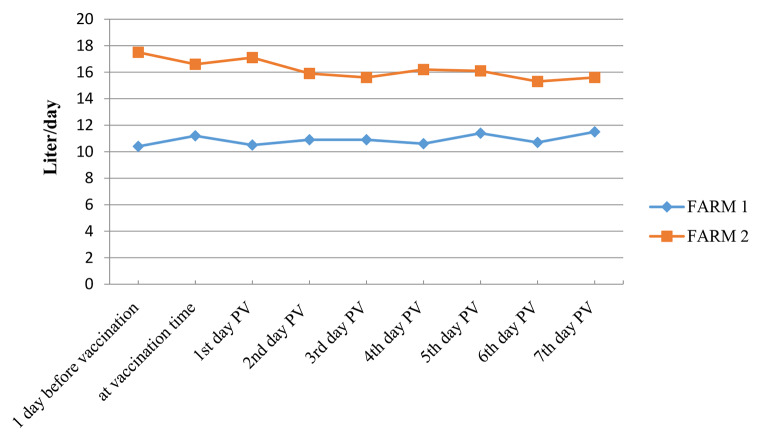
**Average milk yield monitored after vaccination in two cattle farms in Vietnam. Farm-1** (n = 30): average milk yield tended to increase slightly after vaccination (from 10.4 L/day at 1 day before vaccination to 11.5 L/day at 7th day PV). **Farm-2** (n = 35): average milk yield of vaccinated cows decreased slightly after vaccination (from 17.5 L/day at 1 day before vaccination to 15.6 L/day at 7th day PV). PV: Post-vaccination

In addition, the physical activity or health index (restlessness, discomfort, lying, and standing) and milk yield showed limited variations during the first seven days PV. Statistically, the mean health index for the two farms was not significantly different during the first seven days PV (p = 0.80), which was also observed for the milk index (p = 0.45) as shown in Tables 2 and 3; Figs. 3 and 4.

**Table 3:** Mean milk indices in two farms (n = 65 animals) ± standard deviation (SD)

	Before vaccination	At vaccination	1 day PV^※^	2 days PV	3 days PV	4 days PV	5 days PV	6 days PV	7 days PV
Average	13.95	13.9	13.8	13.4	13.25	13.4	13.75	12.95	13.55
± SD	5.02	3.82	4.67	3.54	3.32	3.96	3.32	3.32	2.90

^※^PV: Post-vaccination

#### Efficacy

In the PI experiment conducted in Egypt, the difference between the wild virus titer in vaccinated and non-vaccinated animals was 3.5 log_10_ ID_50_, being one log_10_ higher than the required reference value (> 2.5 log_10_) (WOAH, 2022b). The control animals’ inoculation sites all developed edematous swellings, but the replicas that received the most diluted inocula showed little to no reaction. Within 24 h, the vaccinated animals developed initial hypersensitivity reactions at the sites of inoculation, which quickly diminished.

In the challenge experiment conducted in Vietnam, the vaccinated animals showed a mild rise in temperature (0.5 − 0.9 ºC), and small swellings at the site of injection (1 cm diameter) that disappeared within few hours. The three control animals showed fever (1-1.5 ºC higher than normal) and severe LSD symptoms (swelling diameter between 3.5 and 5.0 cm at the injection site) after the challenge.

#### Potency

Baseline antibody levels of unvaccinated animals (Day 0) were equal or less than 0.6 log_10_ using VNT, while in ELISA values < 30 were considered negative, according to the manufacturer’s recommendations.

**Fig. 5:**
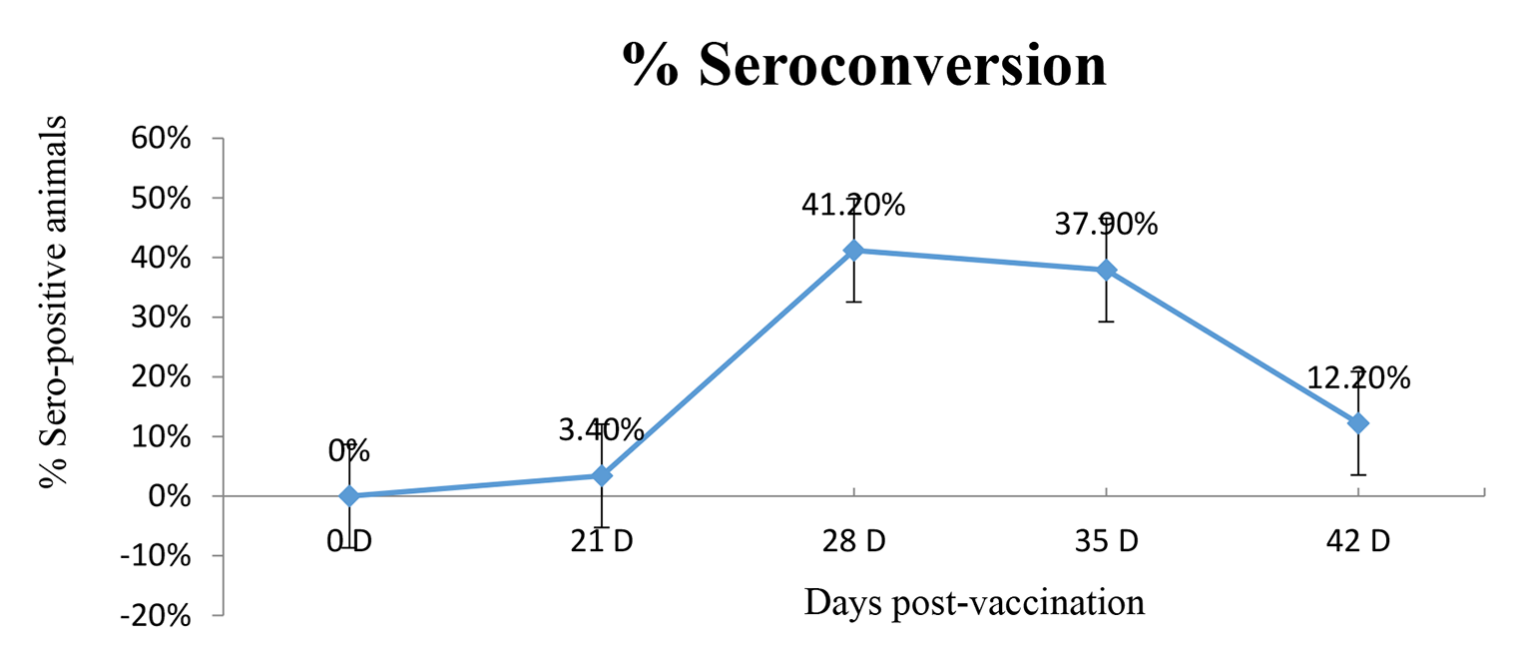
Seroconversion percent of vaccinated animals (n = 65) at different intervals post-vaccination using IDvet Capripox ELISA. (D: days post-vaccination)

Table 4 demonstrates a comparison between the ELISA and VNT results in three vaccinated farms in Egypt (69 cattle in total). ELISA showed a mean positive percent of 51.7 ± 30.6, reflecting a relatively wide range of variation during the monitoring period, as observed in the other experiment conducted in Vietnam (Fig. 5). The corresponding mean for VNT was 78.38 ± 15.18% (Table 4), denoting a higher sensitivity, with titers ≥ 1.2 log_10_ regarded as positive. The relationship between the two tests showed a moderate correlation (r = 0.51). In Vietnam, seroconversion was additionally monitored in two farms by ID-VET ELISA performed simultaneously at three different laboratories (Table 5). The readings came out significantly different between the farms at 28 days PV (p = 0.01) and among labs (lab-2, p = 0.05). In Farm-1, all 3 laboratories showed negative or few positive readings (3.4%) throughout the whole monitoring period. In Farm-2, more positive samples were detected by the three laboratories by 28 days PV (range 34-41.2%, average 37.7%). The average positive samples decreased to 25.03% and 18.2% by days 35 and 42 PV respectively (Table 5).

**Table 4:** Comparison between the results of virus neutralization test (VNT) and ELISA using t-test; the samples were collected from three Egyptian farms at different intervals post-vaccination (n = 69)

Days PV ^†^	VNT^※^ % positive (≥ 1.2 log_10_)	ELISA % positive
28	78.6	50
45	77.4	30
60	57.1	57.1
120	100	100
150	78.6	21.4
Mean ± SD	78.38 ± 15.18%	51.7 ± 30.6

^†^PV: Post-vaccination.

^※^VNT: Virus neutralization test.

**Table 5:** Comparison of ELISA results performed simultaneously at three different laboratories for samples from two farms in Vietnam using t-test (data from Farm-2 were depicted twice: horizontally for comparison with Farm-1 in terms of ELISA results at different intervals post-vaccination and vertically for comparison of results from each lab for the two farms)

Seropositive ELISA results %							P-value (t-test) Farm 1 vs. 2
Days post-vaccination	Farm-1 (n = 29 cows)			Farm-2 (n = 29 cows)			
	Lab-1	Lab-2	Lab-3	Lab-1	Lab-2	Lab-3	
21	0	0	0	0	0	2.9	0.42
28	3.4	3.4	0	34	37.9	41.2	0.01
35	0	3.4	3.4	17.2	37.9	20	0.06
42	0	3.4	3.4	0	37.9	16.7	0.25
	Farm-2 (n = 29 cows)						
21	0	0	2.9				
28	34	37.9	41.2				
35	17.2	37.9	20				
42	0	37.9	16.7				
P-value (T-test) Within each Lab	0.20	0.05	0.10				

### Field study

In the large-scale field studies, PV clinical signs were similar to those reported in the small-scale experiments. Table 6 presents a detailed stratification of physiological conditions of cattle vaccinated in the two farms (n = 4301) in Vietnam and the recorded PV reactions. Results showed that 1.9% (80/4301) of the animals showed signs of hyper-reaction right after vaccination and 0.6% (24/4301) developed small swellings around the injection site and over the body (Table 6). The hyper-reactivity vanished within 3–5 h, PV. Abortion was recorded between two and nine days PV in three animals only out of 867 pregnant animals (0.3%, 3/867) at 142, 197, and 238 days of gestation.

Protection percent against natural LSD infection in the two Vietnamese farms-1 and − 2 reached 98 and 100%, respectively after one year of vaccination. However, in one different small farm, with 50 calves born to unvaccinated dams, the vaccine was safe and protection percent was 88%.

**Table 6:** Stratification of physiological conditions of cattle vaccinated with MEVAC^®^ LSD in two farms in Vietnam: in addition to rate of appearance of post-vaccination reactions

Vaccination	Cattle (head)						Local hyper- reaction		Skin swellings	
	Calves	Young heifers	Pregnant heifers	Milking cows	Dry off cows	Total	No.	%	No.	%
Farm-1	471	234	211	529	132	1577	18	1.1	9	0.6
Farm-2	1196	261	656	165	446	2724	62	2.3	15	0.6
Total	1667	495	867	694	578	4301	80	1.9	24	0.6

Abortion was recorded in 0.3% (3/867) of the pregnant animals.

## Discussion

In this study, the live attenuated MEVAC^®^ LSD vaccine (Neethling strain) was evaluated. In general, the vaccine was well tolerated by all the animals as the impact of vaccination on the general health, ruminal index, and milk yield of vaccinated cattle were almost nonexistent. Data obtained from both experimental and field studies in Egypt and Vietnam showed that the product was safe with no or insignificant PV reactions on the skin (Table 6), health indices (Tables 1 and 2; Figs. 2 and 3) and milk yield (Table 3; Fig. 4). These results suggest a highly satisfactory product, since some other LSD vaccines have shown adverse effects, probably due to higher dose concentration (10^4^ TCID_50_/mL), or insufficient attenuation leading to localized or generalized skin lesions, with detectable vaccine virus in the nodules, blood, and milk. Those vaccines were rejected for lack of safety requirements stipulated by OIE (Bedeković et al., 2018). Other LSD vaccines showed pronounced multiple swellings at the injection sites in 12% of the animals starting at six days PV and lasting for 2–4 days (Katsoulos et al., 2018) or small-sized lumps (< 0.5 cm) in 9% of the animals between days 8 and 18 PV (Calistri et al., 2019; Tekilegiorgis and Tamir, 2019). In our study, the use of a larger number of inoculated animals in the safety test and batch potency (PI test) relative to OIE requirements (WOAH, 2022b) was to assess any risks associated with the vaccine administration. The fewer number of uninoculated controls used, was based on the expectation of no or negligible signs of disease; meanwhile, large groups of animals were vaccinated in the field using the regular dose.

These PV reactions are in agreement with the observations of Tuppurainen et al. (2021) who stated that available LSD vaccines may show variable results in quality, efficacy, safety, and side effects, probably due to type of seed virus used, level of attenuation, dose volume, and titer. Low-level attenuation and heterogenicity of viruses used in vaccination have been associated with incomplete safety and appearance of clinical disease in vaccinated animals (Tuppurainen et al. 2021). However, it has been reported that vaccines based on the Neethling strain are four times more effective than a sheep pox-based vaccine in preventing LSD (Ben-Gera et al., 2015). The relative vaccine effectiveness of one Neethling strain vaccine was 77% and full protection was achieved one month after vaccination, although evidence of effectiveness after two weeks only was demonstrated (Tekilegiorgis and Tamir, 2019).

In the present study, PV comfort was monitored in Vietnam by evaluating the resting behavior and rumination indices of the animals (Vanhoudt et al., 2015). The mean health, milk, and rumination indices values for the two farms were non-significantly different during the first seven days PV. Cows normally ruminate about 450 to 500 min a day (about 8 h), and the observed differences were non-significant, indicating no effect on the digestive system or well-being of the animals. Additionally, the appearance of skin hypersensitivity reactions or small swellings which were associated with vaccine administration, has been mainly local and transient. Unvaccinated animals in experimental studies never showed these signs. Abortion was recorded in 0.3% (3/867) of the pregnant animals by 2–9 days PV at gestation periods < 4 months, suggesting linkage to vaccine administration.

Immunogenicity studies using MEVAC^®^ LSD vaccine showed a mean positive ELISA percent of 51.7 ± 30.6, while the mean positive titers by VNT (≥ 1.2 log_10_) was 78.38 + 15.18% (Table 4). This may indicate a higher VNT sensitivity compared to ELISA, though the results were fluctuating and the correlation between the results of the two tests during the observation period was moderate (r = 0.51). Likewise, one study reported VNT antibodies in 50% of the vaccinated cattle (Hamdi et al., 2020), while another study reported a lower value of 34% (Samojlović et al., 2019). ELISA readings were comparable with VNT, being positive in 30% of the sera and the Kappa Index of Inter-rater Reliability (K) between the two tests was 0.8–0.9 (Samojlović et al., 2019). However, the authors included some VNT titers lower than 1.2 log_10_, and they preferred performing VNT over PI test of OIE, being *in vitro*, easier, and cheaper (Samojlović et al., 2019). Overall, the observed low serological responses (VNT or ELISA) have been explained by the significant role of cellular immunity in the protection against the disease after vaccination (Norian et al., 2019; Varshovi et al., 2017).

In Vietnam, seroconversion was evaluated by ID-VET ELISA only and, interestingly, the results from the three different laboratories came out significantly different at 28 days PV (p = 0.01), either because of inappropriate timing of sample collection in relation to detectable antibody levels or inconsistencies in the method. For instance, in farm-1, all three laboratories showed negative ELISA readings after vaccination and few animals (3.4%) were positive. In farm-2, considerably higher results were obtained from all three laboratories at days 35 and 42 PV. Earlier, Milovanović et al. (2019) showed that Capripoxvirus-specific antibodies were detected by 46 to 47 weeks after vaccination in only 33.77% of the animals using ELISA, while VNT was positive in 35.06% of them. It appears that lab-2 demonstrated significantly higher values in farm-2 compared to farm-1 (p = 0.05). For this reason, the challenge test was recommended to confirm protection (Gari et al., 2015), since studies to evaluate the cell-mediated immunity against LSD are insufficient or un-straightforward (Abutarbush and Tuppurainen, 2018). Previous researchers reported that the number of animals with antibodies against LSDV decreased with time after vaccination (Samojlović et al., 2019), in agreement with our results (Tables 4 and 5). When the vaccine efficacy/effectiveness was measured by calculating the risk of disease among vaccinated and unvaccinated cattle and the percent reduction in risk of disease among vaccinated and unvaccinated animals was determined, protection by MEVAC^®^ LSD was estimated to be 98 and 100% in two farms in Vietnam and was 88% in a third small farm containing 50 calves born to unvaccinated dams. This lower value may be attributed to the vaccination of calves at one month of age before the full maturation of the immune system. Normally calves born to immunized cows will have a passive immunity that persists for about three to four months (Tuppurainen et al. 2021) and vaccination is administered after this period to avoid neutralization by maternal immunity through colostrum (Agianniotaki et al., 2018).

The obtained results show that MEVAC^®^ LSD vaccine produced minor clinical signs, with around 0.6% of the animals exhibiting minor skin inflammatory reactions (< 2 cm diameter nodules) in the field. Those lesions disappeared within few hours PV in the absence of fever or change of appetite. Abortion was recorded in 0.3% (3/867) of the pregnant animals. Vaccinated calves were resistant to challenge, and PI value was 3.5 log_10_.

The majority of the statistical methods used in this study are descriptive, which is one of its limitations; however, the controlled experiments performed (i.e., safety, challenge, and PI) support the conclusions drawn from the descriptive statistics. Furthermore, the large number of animals monitored in the field study could add to the evidence. Overall, our results promise a new safe and potent LSD vaccine.

## Electronic supplementary material

Below is the link to the electronic supplementary material.

Supplementary Material 1

Supplementary Material 2

## Data Availability

Data supporting the results are available on request from the authors.

## Declarations

### Supplementary information

Supplementary files 1 and 2 are showing the ScienSano report, containing information on identity and molecular characterization of the virus used to produce the vaccine.
