# Supplementary material for: Emergency vaccination of cattle against lumpy skin disease: Evaluation of safety, efficacy, and potency of MEVAC® LSD vaccine containing Neethling strain
Source: Vet Res Commun. 2022 Dec 3;47(2):767–77. doi: 10.1007/s11259-022-10037-2 (PMC9734455; doi:10.1007/s11259-022-10037-2)
Supplement: Supplementary file 1 — Supplementary Material 1 [file 11259_2022_10037_MOESM1_ESM.pdf]

Report: Control of Lumpy Skin Disease vaccine batch

Vaccine: Lumpy vac

Batch: 2104210501 (Diluent 2104200601)

Manufacturer: MEVAC

Report: Sciensano-CAPX2104
Report date: 07-07-2021
Report generated by: Wannes Philips, Laetitia Aerts, Andy Haegeman, Ilse De Leeuw, Kris De Clercq

Sciensano · Rue Juliette Wytsmanstraat 14 · 1050 Brussels · Belgium
T + 32 2 642 51 11 · F + 32 2 642 50 01 · info@sciensano.be · www.sciensano.be

healthy all life long

Introduction

Samples were received at Sciensano on June 9th 2021 from MEVAC in Egypt. Analysis was performed at the request of Dr Momtaz Wasfy.

Vaccine Information

**Tenderer**	MEVAC
**Commercial name**	MEVAC LSD
**Name and address of manufacturer**	MEVAC<br>2nd industrial are<br>El-Salhya El-Gdeda<br>El-Sharkya<br>Eqypt
**Manufacturer's batch number(s) appearing on package and other identification numbers associated with this batch**	Batch N°: 2104210501
**Batch number of diluent and expiry date**	Sterile diluent batch N°: 2104200601; expiry date as listed on the CoA 19/04/2023
**Type of container**	Glass vial with Freeze dried vaccine + vial 10 ml diluent
**Number of doses per container**	10
**Production date**	21/04/2021
**Expiry date**	20/10/2022
**Total number of containers received**	3
**Date of receipt**	09/06/2021
**Date of receipt of information on the provided vaccine batch**	24/05/2021

healthy all life long

Test Results

1. Identity of the vaccine strain

1a. Lumpy skin disease virus vaccine strain

Method	Result	Reference
DIVA PCR	Positive for vaccine LSDV strain<br>Negative for wild-type LSDV	Agianniotaki *et al*. 2017 Journal of Virological Methods; 249:48-57

Comment: The reconstituted vaccine contains genome of an LSDV vaccine strain that is differentiable from wild-type LSDV; the reconstituted vaccine does not contain detectable amounts of wild-type LSDV genome, following the methodology of Agianniotaki *et al*. 2017.

1b. Lumpy skin disease virus phylogenetic analyses Method

Method	Fragment name	Fragment length sequenced	reference
Sequencing Primerpair 76013F-76627R	F1	614	In-house
Sequencing Primerpair 109389F-110139R	F2	750	In-house
Sequencing Primerpair 51491F-52246R	F3	755	In-house
Sequencing Primerpair 6070F- 6778R	F4	708	In-house
Sequencing Primerpair 17949F - 18688R	F5	739	In-house
Sequencing Primerpair PolF-PolR	Pol	1000	Lamien et al. 2010

Comment: Fragment 2 and fragment Pol were used for phylogenetic analyses using the MEGA X (Kumar et al., 2018)
The evolutionary history for fragment 2 was inferred by using the Maximum Likelihood method based on the Tamura 3-parameter model (Tamura et al., 1992). For the fragment POL, the Hasegawa-Kishino-Yano model (Hasegawa et al., 1985) was used. Model selection was based on the lowest BIC scores (Bayesian Information Criterion) and AICc value (Akaike Information Criterion, corrected).
Initial tree(s) for the heuristic search were obtained automatically by applying Neighbor-Join and BioNJ algorithms to a matrix of pairwise distances estimated using the Maximum Composite Likelihood (MCL) approach, and then selecting the topology with superior log likelihood value. For the Pol region, a discrete Gamma distribution was used to model evolutionary rate differences among sites.
See annex 1 for the phylogenetic ML trees based on F2 and FPol.

healthy all life long

Blast analysis: Percentage of similarity within the sequenced fragments between available sequences of capripox viruses and vaccine strain

**Available sequences of capripox viruses**		Vaccine strain in Batch MEVAC 2104210501					
		**Sequence fragment**					
**Name**	**Accession**	F1	F2	F3	F4	F5	Pol
Lumpy skin disease virus strain Herbivac LS batch 008, complete genome	MK441838.1	100.00%	100.00%	100.00%	100.00%	100.00%	99.91%
Lumpy skin disease virus strain LSDV/Russia/Saratov/2017, complete genome	MH646674.1	100.00%	100.00%	100.00%	100.00%	100.00%	99.39%
Lumpy skin disease virus isolate Cro2016, complete genome	MG972412.1	100.00%	100.00%	100.00%	100.00%	100.00%	100.00%
Lumpy skin disease virus strain Neethling-LSD vaccine-OBP, complete genome	KX764645.1	100.00%	100.00%	100.00%	100.00%	100.00%	100.00%
Lumpy skin disease virus strain Neethling-Herbivac vaccine, complete genome	KX764644.1	100.00%	100.00%	100.00%	100.00%	100.00%	100.00%
Lumpy skin disease virus strain SIS-Lumpyvax vaccine, complete genome	KX764643.1	100.00%	100.00%	100.00%	100.00%	100.00%	100.00%
Lumpy skin disease virus isolate KZ-Kostanay-2018, partial genome	MT992618.1	100.00%	100.00%	100.00%	98.65%	100.00%	99.30%
Lumpy skin disease virus isolate Neethling vaccine LW 1959, complete genome	AF409138.1	100.00%	100.00%	100.00%	100.00%	100.00%	99.91%
Lumpy skin disease virus isolate Kenya, complete genome	MN072619.1	99.83%	98.77%	99.46%	98.65%	99.19%	98.86%
Lumpy skin disease virus strain KSGP 0240, complete genome	KX683219.1	99.83%	98.77%	99.46%	98.65%	99.19%	98.86%
Lumpy skin disease virus strain China/GD01/2020, complete genome	MW355944.1	99.83%	98.77%	99.59%	100.00%	100.00%	99.83%
Lumpy skin disease virus isolate LSD, complete genome	MW631933.1	99.83%	98.77%	99.46%	98.65%	99.19%	98.78%
Lumpy skin disease virus strain LSDV/Russia/Udmurtiya/2019, complete genome	MT134042.1	99.83%	98.63%	99.46%	100.00%	99.67%	98.86%
Lumpy skin disease virus NI-2490 isolate Neethling 2490, complete genome	AF325528.1	99.83%	98.77%	99.46%	98.65%	99.19%	98.86%
Lumpy skin disease virus isolate LSD-148-GP-RSA-1997, complete genome	MN636843.1	99.66%	99.86%	100.00%	100.00%	100.00%	99.91%

Lumpy skin disease virus isolate LSD-220-2-NW-RSA-1993, complete genome	MN636842.1	99.66%	99.86%	100.00%	100.00%	100.00%	99.91%
Lumpy skin disease virus isolate LSD-220-1-NW-RSA-1993, complete genome	MN636841.1	99.66%	99.86%	100.00%	100.00%	100.00%	99.91%
Lumpy skin disease virus isolate LSD-248-NW-RSA-1993, complete genome	MN636840.1	99.66%	99.86%	100.00%	100.00%	100.00%	99.91%
Lumpy skin disease virus isolate LSD-103-GP-RSA-1991, complete genome	MN636839.1	99.66%	100.00%	100.00%	100.00%	100.00%	99.91%
Lumpy skin disease virus isolate LSD-58-LP-RSA-1993, complete genome	MN636838.1	99.66%	99.86%	100.00%	100.00%	100.00%	99.91%
Lumpy skin disease virus isolate pendik, complete genome	MN995838.1	99.66%	98.77%	99.46%	98.65%	99.02%	98.78%
Lumpy skin disease virus strain Kubash/KAZ/16, complete genome	MN642592.1	99.66%	98.77%	99.46%	98.65%	99.02%	98.78%
Lumpy skin disease virus isolate 155920/2012, complete genome	KX894508.1	99.66%	98.77%	99.46%	98.65%	99.02%	98.78%
Lumpy skin disease virus strain LSDV/Russia/Dagestan/2015, complete genome	MH893760.2	99.66%	98.77%	99.46%	98.65%	99.19%	98.86%
Lumpy skin disease virus isolate Evros/GR/15, complete genome	KY829023.3	99.66%	98.77%	99.46%	98.65%	99.02%	98.78%
Lumpy skin disease virus isolate SERBIA/Bujanovac/2016, complete genome	KY702007.1	99.66%	98.77%	99.46%	98.65%	99.19%	98.86%
Lumpy skin disease virus strain Neethling-RIBSP vaccine, partial genome	MT130502.2	99.66%	98.77%	99.46%	98.65%	99.02%	98.78%
Lumpy skin disease virus strain 210LSD-249/BUL/16, complete genome	MT643825.1	99.66%	98.77%	99.46%	98.65%	99.02%	98.78%
Lumpy skin disease virus NW-LW isolate Neethling Warmbaths LW, complete genome	AF409137.1	99.66%	98.77%	99.46%	98.65%	99.19%	98.78%
Sheeppox virus isolate Turkey vaccine, complete genome	MN072631.1	98.63%	95.09%	96.75%	97.03%	91.25%	94.33%
Sheeppox virus isolate Saudi Arabia, complete genome	MN072630.1	98.63%	N/A	96.61%	97.03%	91.71%	94.33%
Sheeppox virus isolate Pendik, complete genome	MN072629.1	98.63%	N/A	96.75%	97.18%	91.68%	94.33%
Sheeppox virus isolate Saudi Arabia vaccine, complete genome	MN072627.1	98.63%	95.23%	96.61%	N/A	95.01%	N/A
Sheeppox virus isolate Abu Gharib, complete genome	MN072626.1	98.63%	95.23%	N/A	N/A	95.01%	N/A
Goatpox virus isolate Turkey, complete genome	MN072622.1	98.63%	97.95%	96.46%	N/A	94.79%	97.04%

Goatpox virus isolate Vietnam, complete genome	MN072621.1	98.63%	97.53%	96.46%	N/A	94.97%	96.95%
Goatpox virus isolate India, complete genome	MN072620.1	98.63%	97.53%	96.46%	N/A	94.81%	96.87%
Goatpox virus strain AV41, complete genome	MH381810.1	98.63%	97.53%	N/A	N/A	94.97%	96.95%
Sheeppox virus strain Jaipur, partial genome	MG000156.1	98.63%	N/A	96.61%	N/A	86.31%	94.25%
Sheeppox virus isolate SPPV-GL, complete genome	KT438551.1	98.63%	95.23%	96.61%	97.17%	91.25%	94.33%
Sheeppox virus isolate SPPV-GH, complete genome	KT438550.1	98.63%	95.23%	96.61%	97.17%	91.41%	94.33%
Goatpox virus strain Gorgan, complete genome	KX576657.1	98.63%	97.95%	N/A	96.74%	94.17%	97.04%
Goatpox virus FZ, complete genome	KC951854.1	98.63%	97.53%	N/A	N/A	95.23%	96.95%
Sheeppox virus isolate Srinagar passage-40 vaccine, complete genome	MT137384.1	98.63%	95.23%	96.75%	97.32%	91.54%	94.33%
Sheeppox virus isolate V293, complete genome	MW167071.1	98.63%	N/A	96.61%	97.33%	91.68%	94.33%
Sheeppox virus isolate V123, complete genome	MW167070.1	98.63%	N/A	96.75%	97.32%	91.54%	94.33%
Sheeppox virus V104, complete genome	MW020571.1	98.63%	N/A	96.75%	97.32%	91.56%	94.33%
Goatpox virus V103, complete genome	MW020570.1	98.63%	97.53%	N/A	N/A	94.81%	96.87%
Goatpox virus G20-LKV, complete genome	AY077836.1	98.63%	97.95%	N/A	96.74%	94.17%	97.04%
Goatpox virus Pellor, complete genome	AY077835.1	98.63%	97.95%	N/A	96.74%	94.17%	97.04%
Sheeppox virus NISKHI, complete genome	AY077834.1	98.63%	N/A	96.75%	97.32%	91.56%	94.33%
Sheeppox virus A, complete genome	AY077833.1	98.63%	95.23%	96.75%	97.32%	91.69%	94.25%

2. Testing for following contaminants

2a. Foot-and-Mouth Disease virus (FMDV)

Method	Result	References
Real-time PCR for FMDV 3D	Negative	In-house based on Vandenbussche et al. 2017. J Virol Methods. 246:90-94 Vandenbussche et al. 2016. PLoS One. 11(10):e0164463
Real-time PCR for FMDV 5'UTR	Negative	

Comment: The reconstituted vaccine does not contain detectable amounts of FMDV genome

2b. Bluetongue virus (BTV)

Method	Result	Reference
Real-time PCR for BTV	Negative	In-house based on Toussaint et al. 2007. J. Virol. Methods 140 (1-2): 115-123; Vandemeulebroucke et al. 2010. J. Virol. Methods 165:76-82

Comment: The reconstituted vaccine does not contain detectable amounts of BTV genome

2c. Rift Valley Fever virus (RVFV)

Method:	Result:	Reference
Real-time PCR for RVFV	Negative	In-house

Comment: The reconstituted vaccine does not contain detectable amounts of RVFV genome

2d. Pestiviruses

Method	Result	Reference
Real-time PCR for Bovine viral diarrhea virus (BVDV)	Negative	In-house

Comment: The reconstituted vaccine does not contain detectable amounts of BVDV genome

Final Comment:
- The identification tests reveal that the vaccine contains an LSDV vaccine strain that is differentiable from LSDV wild-type virus.
- There are no detectable traces of genome of the extraneous agents examined.
