# Supplementary material for: Emergency vaccination of cattle against lumpy skin disease: Evaluation of safety, efficacy, and potency of MEVAC® LSD vaccine containing Neethling strain
Source: Vet Res Commun. 2022 Dec 3;47(2):767–77. doi: 10.1007/s11259-022-10037-2 (PMC9734455; doi:10.1007/s11259-022-10037-2)

Report: Control of Lumpy skin disease vaccine batch
Annex I: Phylogenetic ML tree fragment 2 and fragment Pol

Vaccine: MEVAC
Batch: 2104210501
Manufacturer: MEVAC

Report: Sciensano-CAPX202104
Report date: 07-07-2021
Report generated by: Wannes Philips, Andy Haegeman, Ilse De Leeuw, Kris De Clercq

Sciensano · Rue Juliette Wytsmanstraat 14 · 1050 Brussels · Belgium
T + 32 2 642 51 11 · F + 32 2 642 50 01 · info@sciensano.be · www.sciensano.be
healthy all life long

Phylogenetic ML tree fragment 2

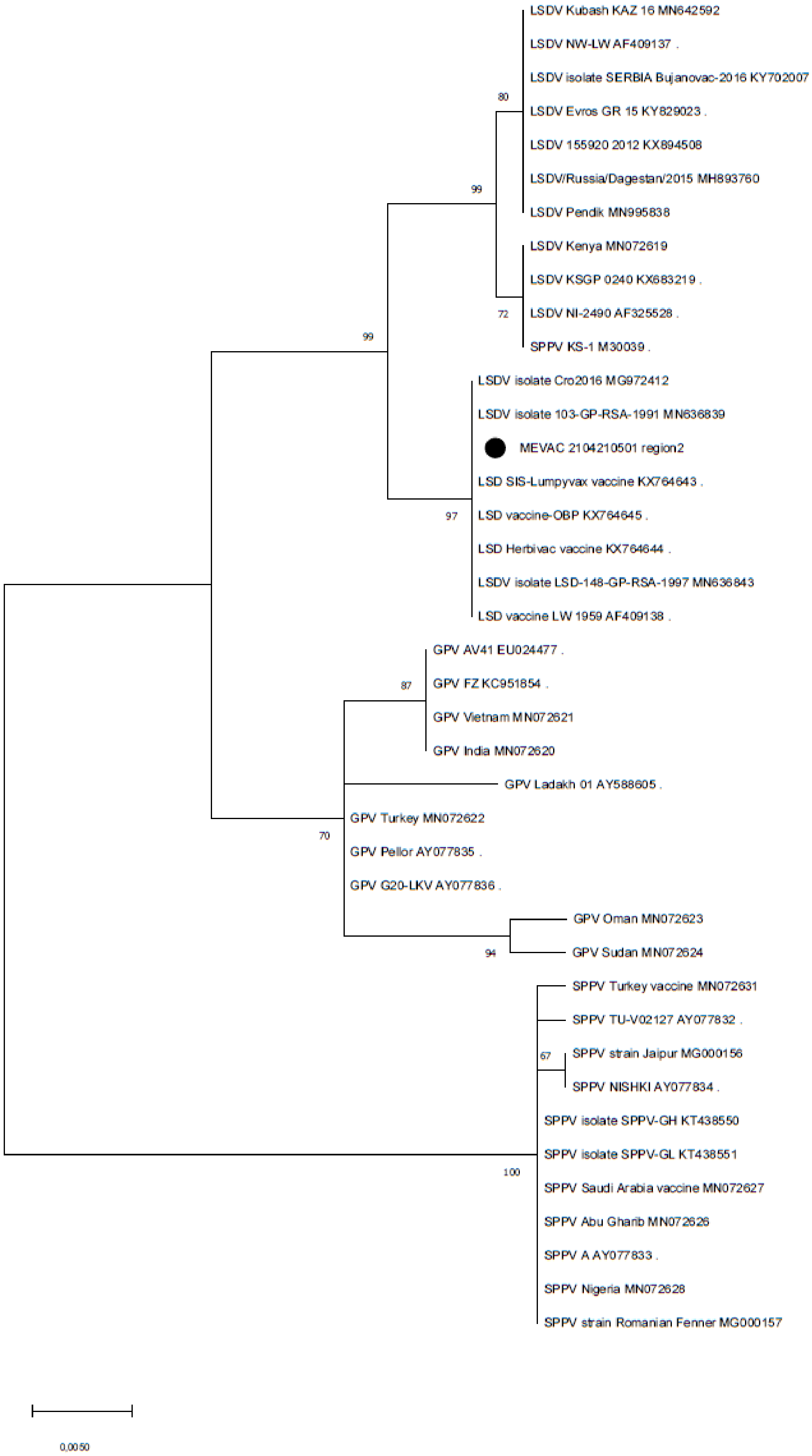

<u>Phylogenetic ML tree fragment Pol</u>

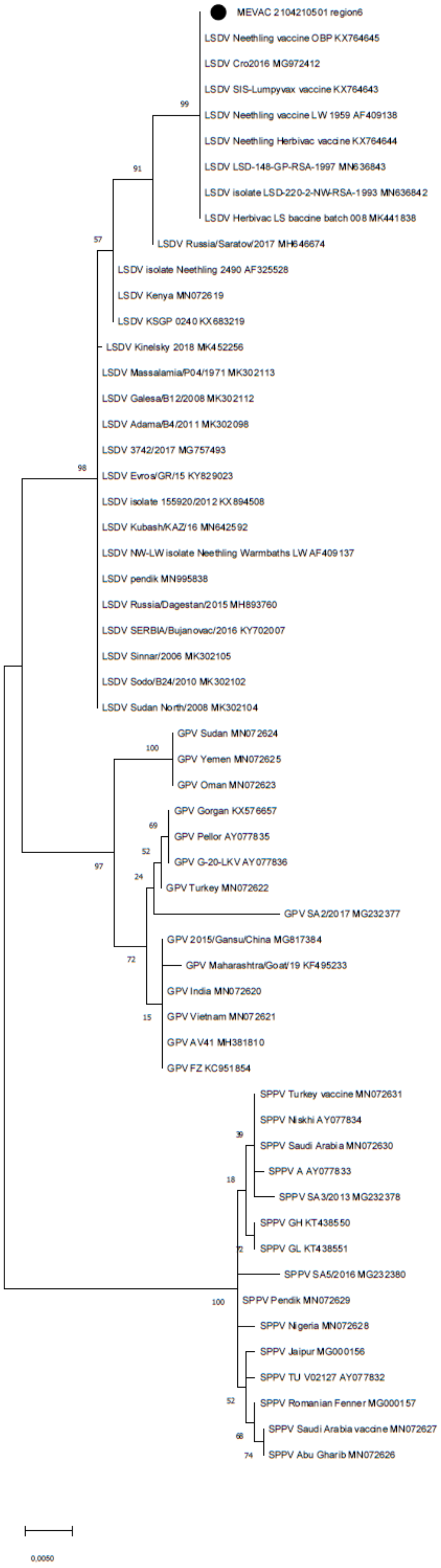

Supplement: Supplementary file 2 — Supplementary Material 2 [file 11259_2022_10037_MOESM2_ESM.pdf]
